# Impact of bisphenol-A on the spliceosome and meiosis of sperm in the testis of adolescent mice

**DOI:** 10.1186/s12917-022-03336-y

**Published:** 2022-07-15

**Authors:** Yongjie Wang, Yanyan Wu, Shilei Zhang

**Affiliations:** 1 Department of Animal Science, Division of Agriculture, University of Fayetteville, Fayetteville, AR 72701 USA; 2 College of Animal Science and Technology, Shihezi University, Shihezi, Xinjiang 832003 China

**Keywords:** Bisphenol-A, Testis, Synaptonemal complex protein 3 (SCP3), DNA damage, RNA-seq, Spliceosome

## Abstract

**Background:**

Bisphenol-A (BPA) has estrogenic activity and adversely affects humans and animals' reproductive systems and functions. There has been a disagreement with the safety of BPA exposure at Tolerable daily intake (TDI) (0.05 mg/kg/d) value and non-observed adverse effect level (5 mg/kg/d). The current study investigated the effects of BPA exposure at various doses starting from Tolerable daily intake (0.05 mg/kg/d) to the lowest observed adverse effect level (50 mg/kg/d) on the testis development in male mice offspring. The BPA exposure lasted for 63 days from pregnancy day 0 of the dams to post-natal day (PND) 45 of the offspring.

**Results:**

The results showed that BPA exposure significantly increased testis (BPA ≥ 20 mg/kg/d) and serum (BPA ≥ 10 mg/kg/d) BPA contents of PND 45 mice. The spermatogenic cells became loose, and the lumen of seminiferous tubules enlarged when BPA exposure at 0.05 mg/kg/d TDI. BPA exposure at a low dose (0.05 mg/kg/d) significantly reduced the expression of Scp3 proteins and elevated sperm abnormality. The significant decrease in Scp3 suggested that BPA inhibits the transformation of spermatogonia into spermatozoa in the testis. The RNA-seq proved that the spliceosome was significantly inhibited in the testes of mice exposed to BPA. According to the RT-qPCR, BPA exposure significantly reduced the expression of *Snrpc* (BPA ≥ 20 mg/kg/d) and *Hnrnpu* (BPA ≥ 0.5 mg/kg/d).

**Conclusions:**

This study indicated that long-term BPA exposure at Tolerable daily intake (0.05 mg/kg/d) is not safe because low-dose long-term exposure to BPA inhibits spermatogonial meiosis in mice testis impairs reproductive function in male offspring.

## Background

Bisphenol-A (BPA) is an important derivative of phenol and acetone, and is widely used in the production of polymer materials such as polycarbonate, epoxy resin, polysulfone resin, polyphenylene ether resin, and unsaturated polyester resin. Additionally, BPA is also used to produce plasticizers, flame retardants, antioxidants, heat stabilizers, rubber antioxidants, pesticides, coatings, and other fine chemical products [1]. It is estimated that the annual global production of BPA is more than 6.8 million tons [2], whereas the annual production of BPA in China reached 1.41 million tons in 2016 [3]. BPA is a ubiquitous environmental endocrine disruptor with pseudo estrogen and antagonistic androgenic effects [4]. Studies have shown that a certain concentration of BPA exposure can seriously affect the development and function of the animal's reproductive system [5–7].

Further, BPA has been detected in landfill leachates, surface water in rivers [8–11], raw milk [12], infusion film bags [13], water bottle [14], canned beer and plastic bottled liquor [15]. Also, BPA was reported to be present in human body fluid samples such as blood, urine [16–18], newborn jaundice [19], saliva, amniotic fluid, and breast milk [20]. Recent studies revealed that BPA is present in indoor dust [21], and its content in the office room is 5 to 10 times more than that in the living room [22]. Increasing amounts of BPA are being released into the atmospheric environment especially in industrial areas and city centers [23].

Studies have shown that BPA exposure produces permanent deleterious effects on humans and animals' reproductive systems and functions [4]. Maternal bisphenol A exposure disrupts spermatogenesis in adult rat offspring [24]. Subjection to BPA causes deleterious effects such as nuclear DNA cleavage in germ cells and apoptosis in the testis in rats or mice [25, 26]. Previous reports suggested that human health may be affected even with exposure to low levels of BPA [4]. Using a mice model to study BPA exposure effects on reproductive organs, RL Jones, SA Lang, JA Kendziorski, AD Greene and KAJEhp Burns [27] adopted the no-observed-adverse-effect-level (NOAEL) and the low-observed-adverse-effect-level (LOAEL) exposure levels. The NOAEL for BPA is 5 mg/kg/day, and LOAEL is 50 mg/kg/day suggested by US Environmental Protection Agency [17]. However, the detailed mechanism of BPA toxicology remains to be elucidated. BPA exposure via placenta first, and lactation and drinking water later, affected the body weight gain in male offspring at 45 postnatal days and the first round of spermatogenesis [28].

Synaptonemal complex protein 3 (Scp3) is a structural component of the synaptonemal complex, which was essential for meiosis [29]. Deleting the SCP3 gene would inhibit synaptosome formation and adversely influence the functions of DNA repair and recombinant proteins, resulting in the failure of meiosis and infertility in mice and humans [30, 31]. Transcriptomic sequencing is often applied to differential gene screening and functional annotation [32]. Current methods for studying transcriptomes are primarily relying on sequencing techniques. In comparison with traditional analytical techniques, transcriptome sequencing analysis will obtain more comprehensive data [33].

We hypothesize that the low-level exposure to BPA, even below NOAEL level, would still be not safe, and it would impact the testis development in male mice offspring. The objective of this study was to evaluate the effects of BPA exposure below LOAEL and NOAEL levels through the placenta, lactation, and drinking water on testis development in male mice offspring by testing the histopathological changes of the testis, sperm quality, expression of SCP3 protein in spermatogenic cells, and by analyzing the testicular transcriptome data.

## Results

### BPA contents in serum and testicular tissues

The measurement results (Table 1) were conducted according to the manufacturer’s instructions and considered credible when the linear regression coefficient was *R*^2^ ≥ 0.92. The coefficient *R*^2^ measured in our study was 0.9985, implying credible results. The results showed that BPA content in the testis of PND 45 male mice was significantly higher than the control group (1.23 ± 0.12) when the BPA exposure dose was at or higher than 20 mg/kg/d (2.17 ± 0.12 in group F and 2.67 ± 0.20 in group G; *P* < 0.05). Further, BPA content in serum reached significantly higher (in groups E, F, G) level than the control group when the BPA exposure dose was at or higher than 10 mg/kg/d (*P* < 0.05). Results indicate that BPA at higher doses can enter the testis.

**Table 1:** BPA content in serum and testis

Groups (*n* = 5)	Serum (ng/mL)	Testis (ng/g)
Control group	1.19 ± 0.10^a^	1.23 ± 0.12^a^
BPA 0.05 mg/kg/d	1.20 ± 0.08^a^	1.30 ± 0.09^a^
BPA 0.5 mg/kg/d	1.37 ± 0.08^ab^	1.35 ± 0.14^a^
BPA 5 mg/kg/d	1.46 ± 0.11^ab^	1.48 ± 0.13^a^
BPA 10 mg/kg/d	1.64 ± 0.16^b^	1.60 ± 0.12^a^
BPA 20 mg/kg/d	2.18 ± 0.13^c^	2.17 ± 0.12^b^
BPA 50 mg/kg/d	2.72 ± 0.12^d^	2.67 ± 0.20^c^

Results are expressed as mean ± standard error. Different superscript letters (a, b, c, d) in each column indicate significant differences (*P* < 0.05)

### Weight of testis and testicular organ index

The testicular organ index of group G (7.23 ± 1.15) was significantly increased (*P* < 0.05) compared with the control group (5.20 ± 0.47). No significant differences were observed between BPA exposure groups B-F and the control group. This means BPA at 50 mg/kg/d elevated testicular organ index. See Table 2.

**Table 2:** Weight of testis and testicular organ index

Groups (*n* = 5)	Organ (mg)	Body Weight (g)	Organ index (mg/g)
Control group	191.43 ± 4.34^a^	37.29 ± 2.74^a^	5.20 ± 0.47^a^
BPA 0.05 mg/kg/d	191.73 ± 4.33^a^	36.63 ± 1.94^a^	5.25 ± 0.20^a^
BPA 0.5 mg/kg/d	195.03 ± 14.92^a^	37.36 ± 1.17^a^	5.20 ± 0.24^a^
BPA 5 mg/kg/d	205.43 ± 14.57^a^	39.67 ± 2.42^a^	5.20 ± 0.43^a^
BPA 10 mg/kg/d	207.20 ± 22.16^a^	36.59 ± 0.51^a^	5.68 ± 0.67^ab^
BPA 20 mg/kg/d	243.97 ± 13.70^ab^	38.43 ± 2.89^a^	6.38 ± 0.31^ab^
BPA 50 mg/kg/d	303.30 ± 64.05^b^	41.29 ± 1.99^a^	7.23 ± 1.15^b^

Results are expressed as mean ± standard error. Different superscript letters (a, b) in each column indicated significant differences (*P* < 0.05)

### H&E staining of testis

**Fig. 1:**
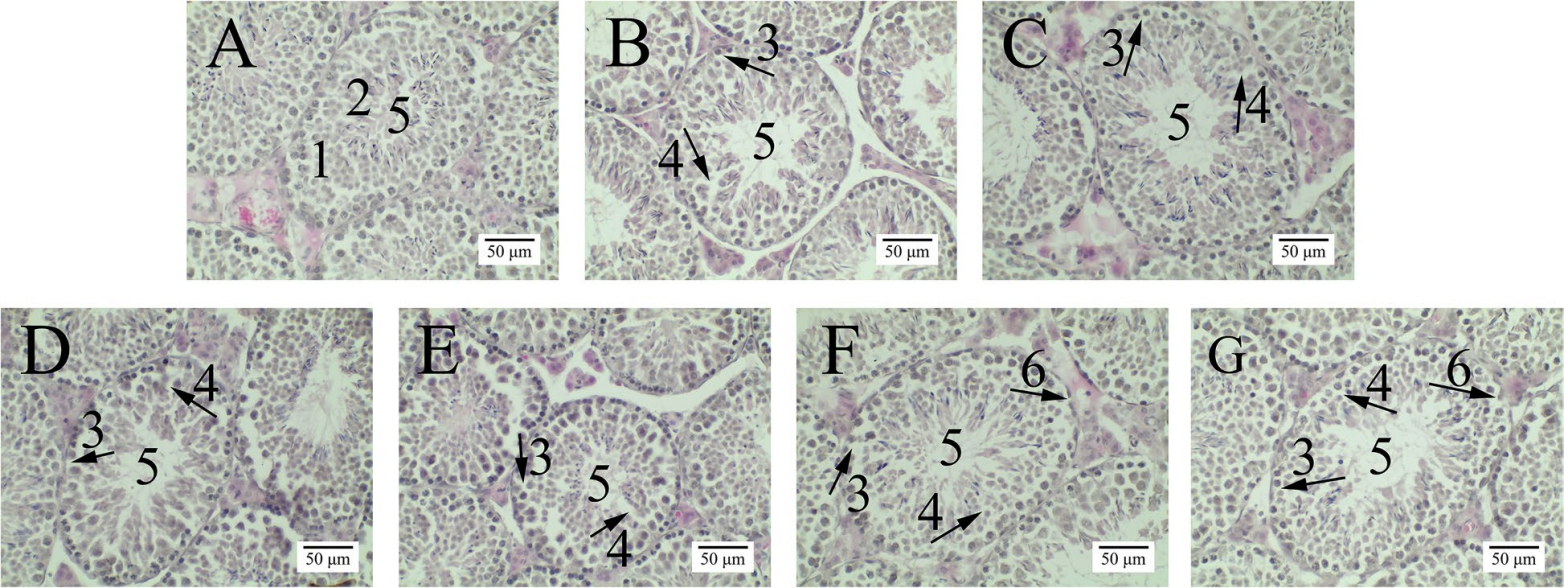
Testicular histopathological changes. Note: **A** the Control group; showing normal morphology of closely packed seminiferous tubules. **B** BPA 0.05 mg/kg /d group; **C** BPA 0.5 mg/kg/d group; **D** BPA 5 mg/kg/d group; **E** BPA 10 mg/kg/d group; **F** BPA 20 mg/kg/d group; **G** BPA 50 mg/kg/d group. 1, indicates the basal lamina; 2, the spermatogonia; 3, spaces between the basal lamina and the spermatogonia; 4, abnormal spaces in-between spermatogenic epithelium spermatogonia; 5, Lumen; 6, incomplete basal lamina. *n* = 13

The spermatogenic epithelium spermatogonia and basal lamina were intact or remained compact in the control group (Fig. 1, A, marked by 1 and 2). The spermatogenic epithelium became loose, and the lumen of seminiferous tubules enlarged when the BPA exposure dose was at or higher than 0.05 mg/kg/d (Fig. 1, B-G, marked by 4 and 5). Noticeable various spaces between the basal lamina and the spermatogonia were observed in BPA exposure groups (Fig. 1, B- G, indicated by 3). The basal lamina of PND 45 mice testis became incomplete (Fig. 1, F-G, marked by 6).

The seminiferous tubule cavities and epithelial height of testis were measured using Image J. The results in Table 3 showed the lumen was enlarged significantly (*P* < 0.05) when BPA doses were at or higher than 0.05 mg/kg/d (50.23 ± 3.54) compared with the control group (37.26 ± 5.32). BPA exposure at or higher than 0.5 mg/kg/d (37.03 ± 2.10) reduced the epithelial height significantly (*P* < 0.05) compared with the control group (52.39 ± 2.83).

**Table 3:** Seminiferous tubule cavities and epithelial height of testis

Groups (*n* = 13)	Lumen diameter (μm)	Epithelial height (μm)
Control group	37.26 ± 5.32^a^	52.39 ± 2.83^e^
BPA 0.05 mg/kg/d	50.23 ± 3.54^b^	48.41 ± 2.95^de^
BPA 0.5 mg/kg/d	62.58 ± 3.10^ cd^	37.03 ± 2.10^abc^
BPA 5 mg/kg/d	69.41 ± 2.62^d^	41.37 ± 1.60^bc^
BPA 10 mg/kg/d	62.69 ± 3.65^ cd^	42.90 ± 2.58^ cd^
BPA 20 mg/kg/d	54.64 ± 4.28^bc^	34.10 ± 1.41^a^
BPA 50 mg/kg/d	64.28 ± 1.90^ cd^	35.01 ± 1.57^ab^

"Lumen diameter" is the diameter of the hollow space in the seminiferous tubules of the testicular HE stained image measured by Image J software; and "Epithelial height" is the thickness of the seminiferous tubules. Results are expressed as mean ± standard error. Different superscript letters in each column (a, b, c, d, e) indicate significant differences (*P* < 0.05)

### Expressions of Scp3 in spermatogenic cells

**Fig. 2:**
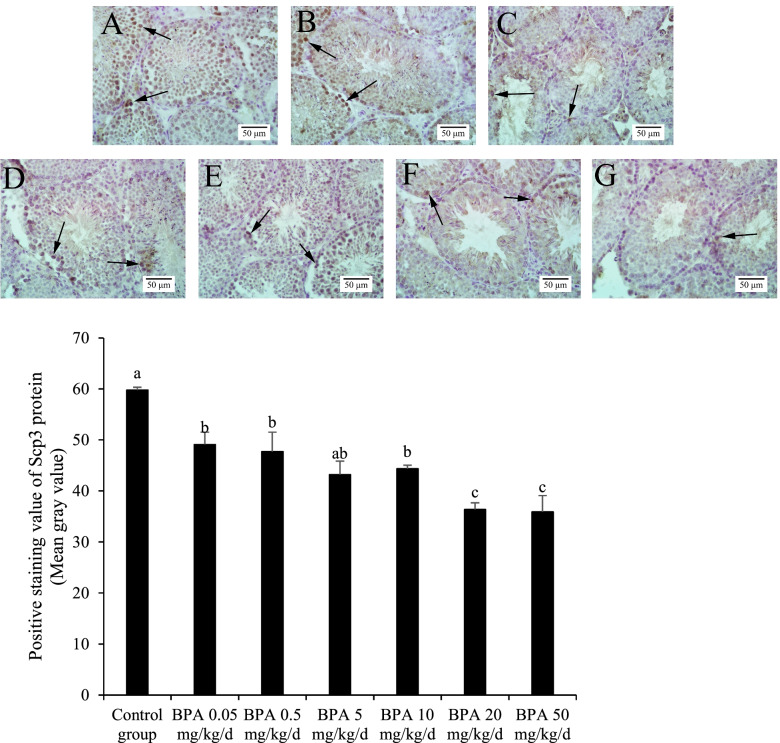
Scp3 expression in pachytene spermatocytes of different BPA groups. Note: Groups **A** was the control group. Groups **B**-**G** were 0.05, 0.5, 5, 10, 20, 50 mg/kg/d BPA groups. The arrows indicate positive staining of Scp3 protein. *n* = 3. The data is the average gray value of positive staining in the testis Scp 3 immunohistochemical results measured by Image J software, and the results are expressed as the mean ± standard error. Different superscript letters on the top of each bar (a, b, c) indicate significant differences (*P* < 0.05)

The Scp3 protein of pachytene spermatocytes was determined, and the expression of Scp3 was gradually decreased with the increase of BPA doses. Statistical analysis results revealed that expression levels of Scp3 protein in the bisphenol A exposed groups were significantly lower than those in the control group (Fig. 2). BPA exposure at a low dose (0.05 mg/kg/d) significantly reduced the expression of Scp3 proteins. A significant difference was observed between the BPA 10 mg/kg/d group and the BPA 20 mg/kg/d group or BPA 50 mg/kg/d group, suggesting a higher concentration of BPA causes more severe damages to the spermatogenic cells.

### DNA damages in mice sperm cells of different BPA treatment groups

**Fig. 3:**
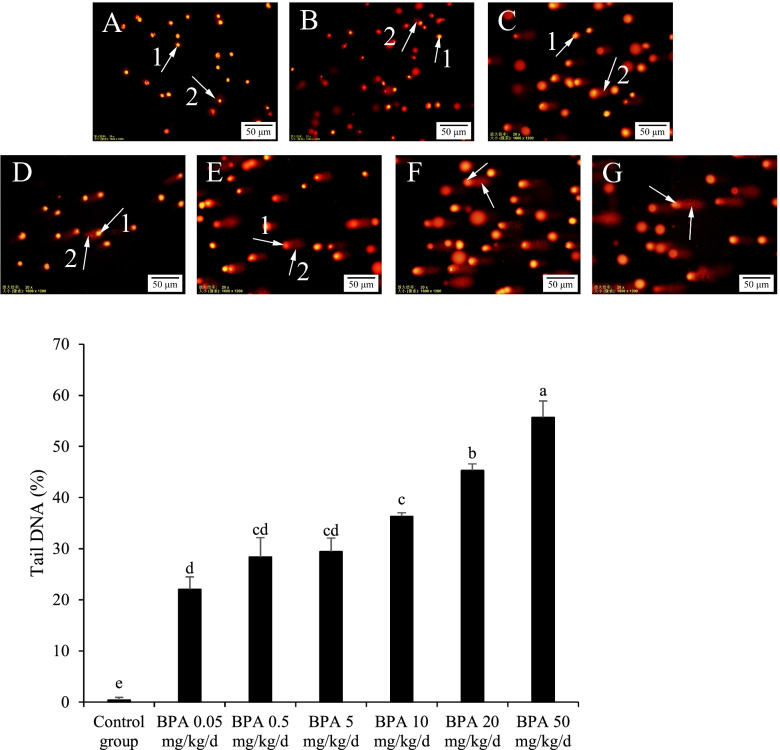
Effect of BPA on mice spermatogonia DNA damage. Note: Group **A** was the control group, groups **B**-**G** were 0.05, 0.5, 5, 10, 20, 50 mg/kg/d BPA groups, respectively. Arrow 1 refers to the nucleus, and arrow 2 refers to the DNA fragments produced by apoptosis. *n* = 200. The results are expressed as the mean ± standard error. Different superscript letters on the top of each bar (a, b, c, d, e) indicate significant differences (*P* < 0.05)

The comet assay visually revealed the DNA damages of the cells through the size of "tail". In this test, the DNA fragments "tail" of mice sperm cells became larger and larger as the increased doses of BPA (Fig. 3). The results implied that the BPA ≥ 0.05 mg/kg/d induced significant damages to the spermatogenic cells.

### Effect of BPA on sperm count and sperm abnormality

**Fig. 4:**
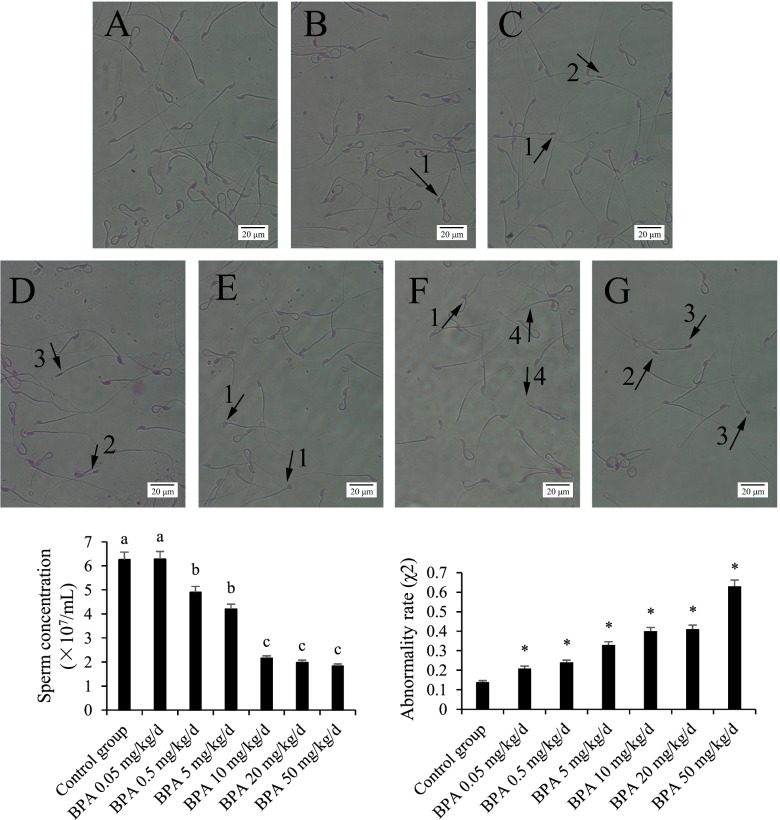
Sperm abnormalities at different BPA doses. Note: Group **A** was the control group, groups **B**-**G** were 0.05, 0.5, 5, 10, 20, 50 mg/kg/d BPA groups, respectively. The sperm count is the number of sperm in the whole field of microscope at 400X magnification after the sperm smear is finished. 1, head malformation; 2, mid-piece deletion; 3, acrosome deletion; 4, tail deletion. The results are expressed as the mean ± standard error. Different superscript letters (a, b, c) or * indicate significant differences (*P* < 0.05)

The sperm abnormality was shown in Fig. 4. The sperm count was significantly reduced in BPA exposure groups (BPA > 0.5 mg/kg/d). The sperm abnormality rate of each BPA exposed group was significantly greater than that of the control group.

### Effects of BPA on testicular gene expression in mice

**Fig. 5:**
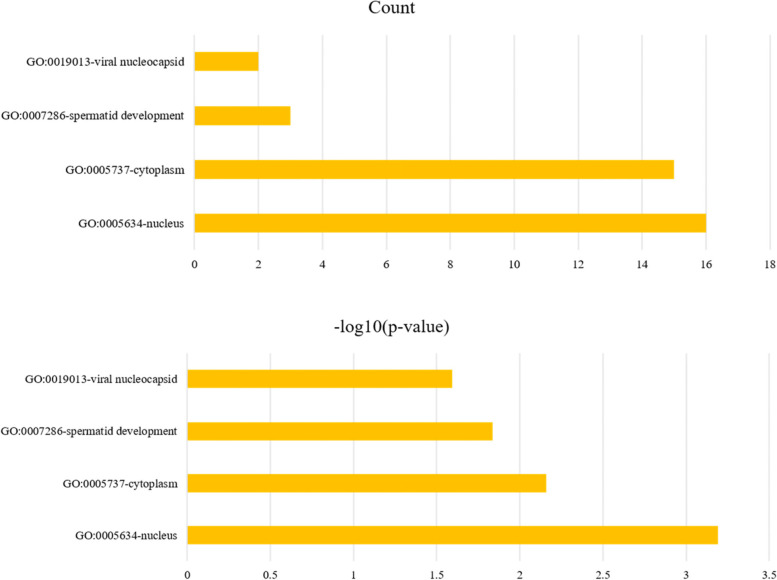
Differential genes GO terms functional annotation clustering. Note: The vertical axis represents the name of the GO categories, and the horizontal axis represents the number or *p*-value of the genes in the GO categories

**Fig. 6:**
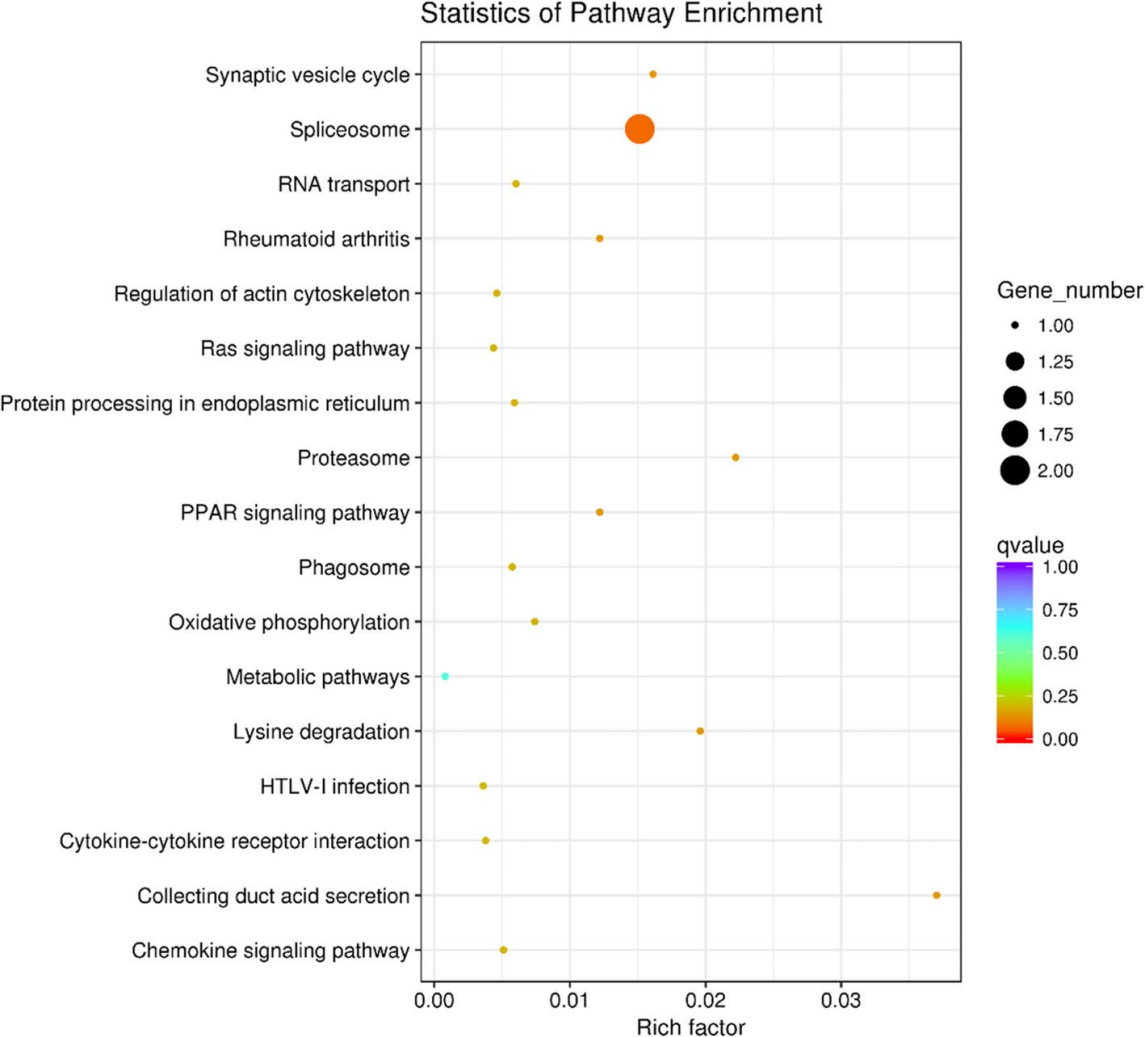
Differential genes KEGG enrichment. Note: The vertical axis represents the name of the metabolic pathway, and the horizontal axis represents the degree of enrichment. The size of the dot indicates the number of differentially expressed genes in this pathway, and the color of the dots corresponds to a different *q*-value. (*n* = 3)

The BPA 50 mg/kg/d group (*n* = 3) and the control group (*n* = 3) were subjected to transcriptome sequencing. The differential expressed genes from the sequencing results were introduced into the GO terms and KEGG database for annotation analysis. The GO terms showed that the differential expressed genes are functionally categorized as genes of viral nucleocapsid, spermatid development, cytoplasm, and nucleus (Fig. 5). The differential genes KEGG enrichment results showed that the shear metabolic pathway was significantly enriched (Fig. 6). The splicing U1 subunit protein C synthesis gene *Snrpc* and the cleavage universal vector component coding gene *Hnrnpu* were significantly down-regulated.

### Real-time PCR verification of the splicesome gene expression

**Fig. 7:**
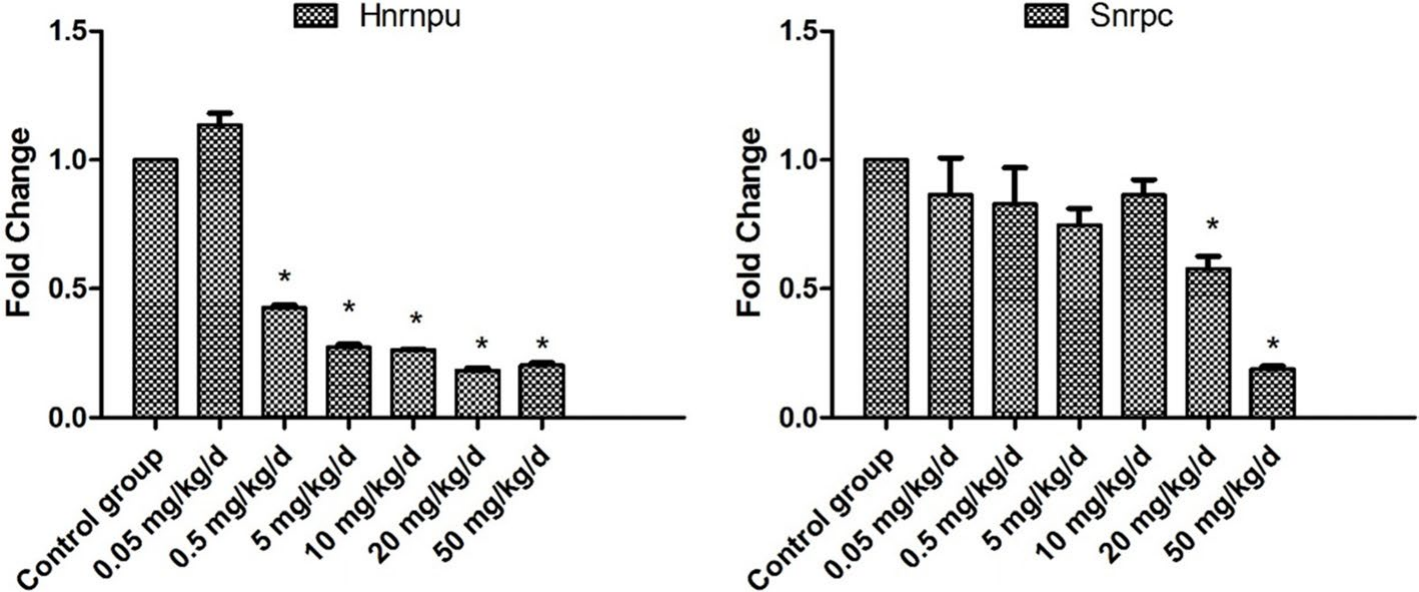
Relative expression levels of *Snrpc* and *Hnrnpu* genes. Note: The ordinate is the differential fold change of *Snrpc* and *Hnrnpu* gene relative to the house-keeping genes, and the abscissa is the seven groups established according to the exposure doses of the BPA in the experiment. The results are expressed as the mean ± standard error. “ *” indicate significant differences (*P* < 0.05). *n* = 3

The expression levels of *Snrpc* and *Hnrnpu* in all 7 groups of testicular tissues were detected by real-time PCR, and the error of RNA yield of different tissues was corrected by *β-actin* and *GAPDH* as house-keeping genes. The results showed that *Snrpc* and *Hnrnpu* decreased with the increase of BPA dosage, which was consistent with the transcriptome sequencing results. However, we can see that *Hnrnpu* is more sensitive to BPA by fluorescence quantitative PCR results. The expression of *Hnrnpu* decreased to less than half of the control group when the BPA dose was 0.5 mg/kg/d or more, while the expression of *Snrpc* decreased to less than half of the control group when the BPA dose was 20 mg/kg/d or more, Fig. 7.

## Discussion

BPA is an environmental endocrine-disrupting chemical widely used in the production of consumer products, with approximately 70% used to produce polycarbonate plastics for a variety of items [34]. Environmental Protection Agency, USA sets no observed adverse effect level (NOAEL) as BPA 5 mg/kg/d and the low observed adverse effect level (LOAEL) as BPA 50 mg/kg/d [27]. Studies showed that BPA could influence testicular development [35]. The contents of BPA in tissues were directly proportional to the exposure dose of BPA [36]. The sexual maturation in male mice was reported to be 36—46 days (mean age: 40.6 ± 0.6) [37]. Therefore, the male offspring were sacrificed at PND 45 for measuring the various parameters indicating testis development.

In this study, the epithelial height of testis was significantly reduced in BPA groups (BPA at ≥ 0.5 mg/kg /d). The lumen of seminiferous tubules showed significant enlargement at the BPA exposure dose ≥ 0.05 mg/kg /d. Next, we tested sperm quality, including sperm count and abnormality rate. The results showed that 0.5 mg/kg /d (lower than NOAEL) BPA significantly reduced the sperm count of the offspring male mice. This means that low BPA exposure, even at NOAEL level, could lead to lower sperm quality, which is consistent with the previous report [28].

We examined the expression of Scp3 in spermatocytes at the pachytene stage to investigate the cause of the decrease in sperm count. The results showed that significantly reduced expression of Scp3 was observed when BPA exposure was at TDI value (0.05 mg/kg /d). The TDI value of BPA was defined by the European Food Safety Authority [17]. Studies have shown that the lack of Scp3 can lead to impaired spermatogonial meiosis, leading to apoptosis [38]. To further validate the test results, we used the comet assay to determine the apoptosis of spermatogenic cells in the testes. The results showed that the BPA exposure dose at 0.5 mg/kg/d (lower than NOAEL) resulted in apoptosis of germ cell significantly greater than the control group (*P* < 0.05).

By GO terms and KEGG enrichment analysis of significantly differentially expressed genes, we found that the splicing function of mice testis was affected by the ingestion of BPA. The spliceosome is a ribonucleoprotein complex (snRNP) for removing pre-mRNA introns and converting them into mature mRNA. The snRNP was composed of five subunits (U1, U2, U4, U5, and U6) and more than 150 structural proteins [39]. In order to initiate the first step of splicing, it is needed to combine the U1 snRNP and universal protein components with the 5' of mRNA [40]. In our present study, the heterogeneous nuclear ribonucleoprotein U (*Hnrnpu*) and the U1 small nuclear ribonucleoprotein C (*Snrpc*) were significantly down-regulated. The *Snrpc* is an essential component for the U1 subunit of the spliceosome, while *Hnrnpu* is an essential subunit for the hnRNPs, a common component protein of the spliceosome. The initial step in splicing is to make the 5' splicing end of immature mRNA bind to U1 snRNP by the common component. Down-regulation of *Snrpc* and *Hnrnpu* makes the first step of post-transcriptional modification of mRNA impossible. Studies have shown that splice-associated proteins are highly expressed in spermatogonia, and alternative splicing is critical for mitotic to meiotic transition in mouse spermatogenesis [41]. RT qPCR results further confirmed that exposure to BPA reduced the expression of *Snrpc* and *Hnrnpu* in the testis, and *Hnrnpu* is more sensitive to BPA than *Snrpc*. Down-regulations of *Hnrnpu* and *Snrpc* made the first step of splicing impossible. Blocking the function of the spliceosome may be an important pathway for BPA affecting testicular development. In conclusion, the current study results revealed that exposure to low dose BPA (at NAOEF or even at TDI) adversely affected testicular development by inhibiting SCP3 protein expression and down-regulation of *Snrpc* and *Hnrnpu* expressions in the testis.

## Conclusions

The current study investigated the effects of BPA exposure at 0.05, 0.5, 5, 10, 20, 50 mg/kg/d on the development of testes in male mice offspring. The results showed that exposure to BPA significantly increased BPA contents in testis at BPA ≥ 20 mg/kg/d and serum at BPA ≥ 10 mg/kg/d of PND 45 mice. BPA exposure at 0.05 mg/kg/d (TDI) caused damages in spermatogenic cells and seminiferous tubules. Subject to BPA at a low dose (0.05 mg/kg/d) significantly reduced the expression of Scp3 proteins and elevated sperm abnormality, suggesting BPA inhibits the transformation of spermatogonia into spermatozoa in the testis. Sperm cells became apoptotic when BPA dosages were at ≥ 0.5 mg/kg/d. BPA exposure significantly inhibited spliceosome and significantly reduced the expression of *Snrpc* (BPA ≥ 20 mg/kg/d) and *Hnrnpu* (BPA ≥ 0.5 mg/kg/d). This study has fully demonstrated that low-dose long-term exposure to BPA inhibits spermatogonial meiosis and impairs the reproductive function of testis in male mice.

## Materials and methods

### Reagent and animal treatments

BPA stock solution at 50 mg/ml was prepared using solid BPA (purity ≥ 99%, Sigma, US), and stored at 4℃. When used, the stock solution was fixed to the desired concentration with distilled water containing 0.5 mol/L NAOH and 25% ethanol.

The experiments were conducted with 8-week-old Kunming mice weighing 20 ± 2 g, supplied by the Beijing Sibefu Experimental Animal Center (ID number: SCXK, Beijing 2016–0002). Two females were caged overnight, with one male after one week of acclimatization. Confirmation of a vaginal plug in the following morning was designated as gestation day 0 (GD 0). Pregnant mice were then randomly divided into 7 groups of 20 mice each and given different doses of BPA by drinking water from GD 0 to the end of the lactation period. Group A was the control group in which the mice received BPA at 0.00 mg/kg/d; Mice in groups B-G were treated with BPA at 0.05, 0.5, 5, 10, 20, 50 mg/kg/d in drinking water, respectively. After weaning at postnatal day 21 (PND 21), the F1 male mice continued to drink BPA at the same dosages as their mothers until day 45 (PND 45). The total BPA exposure duration was 63 days. To ensure the accurate intake amount of BPA solution, water daily intake by each group of mice was accurately measured. The concentration of BPA solution was adjusted accordingly, as described by Zhang and his colleagues (2019). Animal using procedures were approved by the Animal Welfare Committee of the Agricultural University of Hebei, China (approval No. IACECHEBAU20171017).

### Measurement of BPA contents in serum and testicular tissues

BPA levels in serum and testes homogenates of the F1 mice were detected using the BPA assay kit (Shanghai Runyu Biotechnology Co., Ltd., Lot No: RY-12919) following the manufacturer’s instructions. Briefly, serum or testicular homogenate of 50 μl was added to the ELISA plate in duplicates and incubated at 37 °C for 30 min. Then 50 μl enzyme-labeled reagents were added after washing and incubated at 37 °C for 30 min. The optical density (OD) values were read at 450 nm using a plate reader (H4MFPTAD, BioTek).

### Weight of testis and testicular organ index

The testicular index was calculated as the percentage(%) of weight of both testes (mg) to the body weight (g).

### H&E staining of testes

Testes samples were fixed with 4% paraformaldehyde and embedded in paraffin. 5 μm thickness was prepared and stained with hematoxylin–eosin (H&E) to observe histopathological damages and immunohistochemistry. The number of males used is three and the number of cells/sections assessed per male is three.

### Immunohistochemistry for synaptonemal complex protein 3 (Scp3) in testes

The paraffin sections of testicular samples were xylene-dewaxed and rehydrated with graded ethanol. The sections were then incubated with 3% H_2_O_2_ at 37 °C for 10 min to quench the endogenous peroxidase. After washing with phosphate-buffered saline (PBS) three times, the slides were incubated with bovine serum albumin (BSA,5%) at 37 °C for 10 min to reduce the non-specific staining. After that, the sections were covered with rabbit anti-mouse SCP-3 antibody (bs-3509R, Bioss, China) and incubated overnight at 4 ℃. The sections were then incubated with goat anti-rabbit antibody (bs-0259G-Bio, Bioss, China) at 37 ℃ for 30 min. The sections were covered with horseradish peroxidase-labeled streptavidin (bs-0437P-HRP, Bioss, China) at 37 ℃ for 30 min after being washed three times with PBS. Finally, the sections were washed with PBS and then visualized with 3,3′-diaminobenzidine (DAB; CWBIO). The positive cells were stained in brown color. The negative controls were processed in the same procedure except that the primary antibody was replaced with PBS. The number of males used is three and the number of cells/sections assessed per male is three.

### Spermatogenic cells DNA damage in testes

Testes were chopped and mixed with RMPI 1640 medium (37℃), then pipette 10 μl of the sample and mixed it with 120 μl low melting point agarose (0.5%, 37℃). Then, the mixture was transferred to the glass slide and let to solidify. The finished slides were placed in lysate solution for digestion (4℃, 1 h; Lysate solution: 2.5 mol/L NaCl, 100 mmol/L MTA, 10 mmol/L Tris, aqueous solution, pH10.0, 89 ml; Triton X- Mix 100 1 ml and DMSO 10 ml). After that, electrophoresis (4℃, 1.2 V/cm, 300 mA, 20 min, Running buffer: 300 mM Na OH, 1 mM EDTA, aqueous solution, pH 13.0) was performed. Finally, the slides were stained with ethidium bromide (20 μg/ml) and observed using fluorescence microscopic imaging system (Olympus BX43, US). The tail formed by DNA fragments represents the degree of DNA damage. There are 6 mice in each group, and the two testes of each mouse were used for Single-cell gel electrophoresis, measuring 100 cells per electrophoresis picture. A total of 200 cells were measured per mice.

### Sperm count and abnormality

The cauda epididymis of mice was collected and macerated in 1 mL of phosphate-buffered saline. The obtained suspension was filtered to remove tissue debris. Then the sperm suspension was diluted one thousand times, 10 μl was taken and counted using a haemocytometer to calculate the sperm density in the sperm suspension [42]. In measuring sperm abnormality, the separated cauda epididymis was placed in 1 ml phosphate-buffered saline and minced to obtain the suspension as mentioned above. Then the suspension was filtered through a fine mesh cloth to remove tissue debris. After the staining with 1% eosin Y for 20 min, a suspension drop was transferred onto a clean slide and air-dried. One thousand sperms per animal were scored from each group for the presence of sperm shape abnormalities [43].

### RNA sequencing

Total RNA from the testicular tissues was extracted using Eastep Super Total RNA Extraction Kit (LS1040, Promega, USA). Then it was reversed using the Advantage RT-for-PCR Kit (Code No. 639505, TAKARA, Japan) for the cDNA synthesis. The testis of the BPA 50 mg/kg/d group and the control group were subjected to transcriptome sequencing using the Illumina HiSeq Illumina sequencing platform. The GO categories and KEGG pathways were clustered using the DAVID Functional Annotation Clustering tool [44, 45].

### RT-qPCR for splicesome

All 7 groups of differentially expressed genes in the splicing were verified by RT qPCR. The *β-actin* and *GAPDH* were selected as house-keeping genes. All primers were verified for their amplification efficiency and correlation coefficient R^2^ to ensure the reliability by using 5-points standard curve with 1:10 dilution (Table 4). RT qPCR method: RT qPCR mixture10 μl; forward primer 1 μl; reverse primer 1 μl; cDNA of samples 2 μl (total 100 ng cDNA); ddH_2_O 6 μl. Amplification procedure: 95℃ for 600 s; 95℃ for 30 s, 60℃ for 10 s, 45 cycles. Melting curve program: 95℃ for 10 s, 65℃ for 60 s and then ramped to 97℃ at a rate of 0.2℃/ s, and finally cooled at 37℃ for 30 s.

**Table 4:** Real-time PCR primer sequences

Gene ID		Sequence(5'-3')	GC %	Tm℃	Length (bp)	Amplification efficiency(%)	R^2^
*Snrpc*	F	AACGGCTTGTGGAGCATCA	52.6	62.8	177	101.2	0.994
	R	TGTCTTGTCAATCAGGCTCTGG	50	62.7			
*Hnrnpu*	F	TGCCCAACAGAGGGAACTATAACC	50	64.5	142	97.3	0.993
	R	CCTTGGTGATAATGCTGACTCCA	47.8	63.5			
*β-actin*	F	TCCTTCCTGGGCATGGAGT	57.9	63	104	96.1	0.998
	R	AGCACTGTGTTGGCGTACAG	55	60			
*GAPDH*	F	TGCTGGTGCTGAGAGTATGTGGTG	54.2	64.7	293	104.1	0.995
	R	TCTTCTGGGTGGCAGTGATGG	57.1	62.1			

### Statistical analysis

Immunohistochemistry results were analyzed using Image J, and the results of the comet assay were detected using the comet assay software project (CASP) software. The test data were analyzed with SPSS 26.0 software, and the results were expressed as mean ± standard error (x ± SEM). Data were analyzed by one-way ANOVA and Chi-square test (χ^2^), *P* < 0.05 indicates significant difference.

## Data Availability

The data of this study are available on request from the corresponding authors. The RNA-seq datasets generated and/or analyzed during the current study are available in the NCBI repository GSE198779.
